# Circulating mir-483-5p as a novel diagnostic biomarker for acute coronary syndrome and its predictive value for the clinical outcome after PCI

**DOI:** 10.1186/s12872-023-03387-5

**Published:** 2023-07-18

**Authors:** Yuying Zhao, Xinxing Song, Yanzhuo Ma, Xiang Liu, Yuhong Peng

**Affiliations:** Department of Cardiology, No. 980 Hospital of PLA Joint Logistics Support Force, No 398, Zhongshan West Road, Shijiazhuang, 050082 Hebei China

**Keywords:** Acute coronary syndrome, miR-483-5p, Major adverse cardiovascular events, Diagnostic, Predicts

## Abstract

**Background:**

MicroRNA (miRNA) plays a critical function in the progression of acute coronary syndrome (ACS) and is associated with major adverse cardiovascular events (MACEs) after undergoing percutaneous coronary intervention (PCI). This research was designed to probe the diagnostic accuracy of miR-483-5p in patients with ACS and its predictive value of MACEs.

**Methods:**

118 patients with ACS (40 with unstable angina pectoris [UAP] and 78 with acute myocardial infarction [AMI]) and 75 healthy controls were enrolled. Serum miR-483-5p was detected in the subjects by reverse transcription-quantitative real-time PCR (RT-qPCR). ROC curve and logistic regression models were employed to estimate the diagnosis. Patients were monitored for 6 months after PCI to document the occurrence of MACEs. Kaplan-Meier survival was conducted to explore the predictive significance of miR-483-5p for the MACEs.

**Results:**

Serum miR-483-5p levels were higher in ACS patients and associated with SYNTAX score and Gensini score. miR-483-5p was effective in identifying ACS patients from healthy individuals (AUC = 0.919) and AMI patients from ACS patients (AUC = 0.867), demonstrating a high diagnostic value, proven by logistic regression (OR = 9.664, 95%CI = 4.462–20.928, *P* < 0.001). The prevalence of MACEs during follow-up were 24.58%, and a higher prevalence of MACEs were observed in patients with elevated miR-483-5p (*P* = 0.01). miR-483-5p was also an effective predictor of MACE occurrence (HR = 5.955, 95%CI = 1.928–18.389, *P* = 0.002).

**Conclusion:**

Expression of serum miR-483-5p can be utilized as a non-invasive marker for diagnosing ACS and predicting the onset of MACE after PCI.

## Background

Acute coronary syndrome (ACS) accounts for more than 1 million deaths worldwide each year [1]. The incidence of ACS in China is increasing annually and is predicted to reach 22.6 million patients by 2030 [2]. ACS commonly arises from the rupture or erosion of atherosclerotic plaques in the coronary arteries that supply blood to the heart, resulting in arterial thrombosis and subsequent myocardial ischemia. This is primarily manifested as acute myocardial infarction (AMI) and unstable angina pectoris (UAP) [3]. As a significant burden on global healthcare, ACS has an urgent attack, rapid illness, and heavy mortality rate, and earlier diagnosis and detection can help provide timely management measures for ACS patients [4]. Percutaneous coronary intervention (PCI) is currently the main method for ACS, significantly regaining correct coronary and reducing infarct size. However, 23% of patients exhibited a propensity for major adverse cardiovascular events (MACEs), such as recurrent angina and revascularization [5]. Hence, it is particularly important to identify dependable and consistent biomarkers for the diagnosis and evaluation of MACE.

MicroRNA, as an endogenous one-stranded non-coding RNA molecule, is involved in various biological processes by regulating target genes. Furthermore, miRNA has been proposed as a clinical biomarker for cardiovascular disease owing to its stable presence in various biological fluids (such as blood, serum, urine, and saliva) and its extracellular secretion that can be easily quantified by reverse transcription-quantitative real-time PCR (RT-qPCR). miR-142-3p [6], miR-941 [7], miR-3646 [8], and miR-497-5p [9] and miR-361-5p [10] were identified as promising biomarkers for ACS. miR-483-5p is a mature miRNA consisting of a 22 nucleotide (AAGACGGGAGGAAAGAAGGGAG) located on chromosome 11p15.5. Coronary plaque rupture is most commonly responsible for ACS [11]. Li et al. identified several miRNAs, including miR-483-5p, that exhibited differential expression before and after coronary plaque rupture [11]. Tian et al. demonstrated significant upregulation of miR-483-5p in both plaque arteries and normal coronary arteries via microarray assay [12]. Arrhythmias are a common complication of ACS, and miR-483-5p was reported to be markedly elevated in atrial fibrillation, the most common form of arrhythmia [13].

Herein, the present study aimed to assess the levels of serum miR-483-5p in patients with ACS and investigate its potential as a novel diagnostic biomarker for ACS. Additionally, it explored the predictive value of miR-483-5p about MACE following PCI. A first demonstration is that miR-483-5p expression can be used as a diagnostic marker for ACS and can non-invasively predict the occurrence of MACE after PCI.

## Methods

### Ethical statement

Subjects signed an informed consent form before enrollment. With the approval of the No.980 Hospital of PLA Joint Logistics Support Force Medical Ethics Committee, this research protocol strictly adhered to the Helsinki Declaration principles.

### Study population

Patients aged 40–80 years old who visited the No.980 Hospital of PLA Joint Logistics Support Force for ACS with chest pain diagnosed as UAP, non-ST-elevation myocardial infarction (NSTEMI) and ST-elevation myocardial infarction (STEMI) from January 2018 to June 2019 were included. Inclusion criteria: (1) patients with ACS who met the European Society of Cardiology (ESC) [14] and American College of Cardiology (ACC)[15, 16] diagnostic criteria and had chest pain episodes of less than 24 h; (2) all patients with ACS were first-time episodes; (3) confirmed by coronary angiography with at least 1 coronary artery stenosis with > 75% stenosis (diameter method), requiring PCI treatment; (4) patients with complete clinical data. Exclusion criteria: (1) patients with recent use of immunosuppressants or immune enhancers; (2) combined with other cardiac insufficiencies, hematologic diseases, malignancies, or autoimmune diseases; (3) prior revascularization therapy [either PCI or coronary artery bypass grafting (CABG)]. The final 118 patients with ACS were included in this study, including 40 with UAP and 78 with AMI. The definition of UAP is clinical symptoms, Braunwald’s classification of class IIB and IIIB typical anterior chest pain, and no significant increase in serum creatine kinase concentration. The definition of AMI is characterized by clinical symptoms, coronary angiographic findings, electrocardiogram (ECG) suggestive of new onset, NSTEMI or STEMI, and serum creatine kinase (CK) concentrations were more than two-fold above the upper limit of the normative range. And cardiac troponin I (cTnI) values of more than 0.06 ng/ml were also confirmed for AMI. Furthermore, 75 healthy individuals matching the age and gender of the ACS patients and who were physically examined at the hospital served as controls. diastolic blood pressure (DBP) and systolic blood pressure (SBP) were measured by an Ormon Hem-7136 monitor. cTnI, as well as triglyceride (TG), total cholesterol (TC), low-density-lipoprotein cholesterol (LDL-C), and high-density lipoprotein cholesterol (HDL-C), were assessed by a Roche automated biochemical analyzer. Patient demographics and biochemical data were recorded in Table 1.

**Table 1:** General information of the enroll participants

Parameters	Controls (n = 75)	ACS (n = 118)	*P* values
Age, years	60.12 ± 8.26	58.81 ± 7.96	0.275
BMI, kg/m^2^	25.80 ± 3.77	26.75 ± 4.07	0.107
Gender, male, n (%)	45 (60.00)	68 (57.63)	0.766
Smoking, n (%)	46 (61.33)	64 (54.24)	0.372
SBP, mmHg	127.71 (124.74, 130.68)	129.69 (123.75, 136.62)	0.070
DBP, mmHg	78.90 (72.91, 82.89)	82.17 (77.22, 88.11)	0.002
LDL-C, mmol/L	2.59 ± 0.54	2.81 ± 0.80	0.040
HDL-C, mmol/L	1.36 (1.02, 1.64)	1.35 (0.89, 1.63)	0.278
TC, mmol/L	3.99 ± 0.58	4.12 ± 0.44	0.068
TG, mmol/L	1.48 ± 0.23	1.53 ± 0.56	0.437
Creatine, µmol/L	67.76 (60.35, 77.85)	73.77 (68.48, 78.39)	0.029
WBC, ×10^9^/L	6.68 ± 0.50	7.41 ± 0.71	0.000
UREA, mmol/L	4.92 ± 0.37	4.88 ± 0.51	0.586
NT-proBNP, pg/mL	36.60 ± 6.04	110.59 ± 36.51	0.000
hs-CRP, mg/L	-	5.97 ± 1.39	-
cTnI, ng/mL	-	1.13 (0.05, 2.17)	-
SYNTAX score	-	28.90 ± 10.95	-
Gensini score	-	49.13 ± 26.49	-
Culprit lesion, n (%)			
Left main		1 (0.85)	
Left anterior descending		42 (35.59)	
Left circumflex		29 (24.58)	
Right		46 (38.98)	
Baseline stenosis (%)		94.08 ± 3.87	
MVD, n (%)		73 (61.86)	
Stent diameter (mm)		3.06 ± 0.48	
Stent length (mm)		26.50 ± 4.47	
Aspirin/clopidogrel, n (%)		18 (15.25)	
ACE inhibitor/ARB, n (%)		31 (26.27)	
β-blocker, n (%)		25 (21.19)	
Calcium Channel blocker, n (%)		12 (10.17)	
Stain, n (%)		18 (15.25)	

Note: ACE, angiotensin converting enzyme; ARB, angiotensin receptor blocker; BMI, body mass index; SBP, systolic blood pressure; DBP, diastolic blood pressure; HDL-C, high-density lipoprotein cholesterol; LDL, low-density lipoprotein cholesterol; MVD, multivessel disease; TC, total cholesterol; TG, triglyceride; WBC, white blood cells; hs-CRP, high-sensitivity C-reactive protein; cTnI, cardiac troponin I; Date was presented as mean ± SD, or median (first quartile, third quartile), or N (%)

### Clinical samples collection

5 mL of venous blood was obtained immediately on admission (within 24 h before PCI), and the upper serum was collected by centrifugation at 3000 g for 15 min at 4℃ into RNase-free EP tubes and stored in a -80℃ refrigerator for backup.

### Coronary lesion evaluation and PCI procedure

Quantitative analysis of coronary lesions in ACS patients based on the Syntax score calculator (http://www.syntaxscore.com) and the left-right dominant classification of coronary arteries, lesion location, degree of stenosis, and pathological features based on coronary angiography by two experienced specialists [17]. As the score is higher, the more severe the coronary artery lesion becomes and the worse the outcome. Gensini score was employed to assess the degree of coronary stenosis and severity of atherosclerosis based on prior investigations [18].

PCI was performed in a standard manner, with adequate intraoperative balloon pre-dilation of the target lesion, balloon post-dilation after stent placement, and stents were selected from the new generation of drug-eluting stents, with images showing good stent deployment and less than 5% residual stenosis. Treatment with aspirin and clopidogrel during follow-up.

### Follow up program

After PCI treatment discharge, the researchers followed the patients for 6 months using outpatient, telephone, and readmission visits. Follow-up endpoints were defined as the occurrence of MACEs such as sudden cardiac death, reinfarction, angina, clinically driven target vessel revascularization (including PCI and CABG), and new onset heart failure, and were judged by the investigators based on ECG, ischemic symptoms, and cardiac enzyme levels. Angina was defined as the recurrence of typical angina pectoris after PCI, manifested as retrosternal or precardiac pain at activity or rest, usually lasting no more than 20 min, and the onset was caused by changes in electrocardiogram ST-T and no increase in myocardial injury indexes. Reinfarction was defined as a significant increase in markers of myocardial injury during follow-up, ECG evidence of short ST elevation of more than 1 mm in two or more adjacent, or new left bundle branch blocks, and new pathological Q wave on ECG. Coronary angiography was utilized to determine the location of obstructive lesions in all patients with Reinfarction. New-onset heart failure was defined as the presence of dyspnea from PCI procedures, including exertional dyspnea, terminal and nocturnal paroxysmal dyspnea, signs of pulmonary edema or peripheral edema, ventricular enlargement, and echocardiographic systolic insufficiency.

### Reverse transcription-quantitative real-time PCR (RT-qPCR)

600 µL of Trizol LS was mixed with the serum and left to stand at room temperature. The RNA was precipitated by adding chloroform as well as isopropanol, washed with 75% ethanol after centrifugation, and the RNA precipitate was dissolved in 30 µL of RNA-free water. The purity and concentration of isolated and extracted RNA were assessed using Nanodrop spectrophotometry. The miRNA cDNA synthesis kit (CW2141, Cwbiotech, Beijing, China) was performed at 37℃ for 15 min for miRNA addition (A) tail, followed by synthesis of miRNA cDNA at 42℃ for 50 min and 85℃ for 5 min. After mixing cDNA, primers, and miRNA qPCR Assay kit, amplification reactions were performed in a LightCycler 480 machine (Roche Applied Science). U6 served as an internal control and the relative level of miR-483-5p was obtained after three replicates with the 2^−ΔΔCt^ method.

### Statistical analysis

Kolmogorov-Smirnov was applied to examine the normal distribution of data. Normally distributed variables were illustrated as mean ± SD and analyzed by Student’s t-test, while non-normally distributed continuous data were indicated using median and quartiles [M(Q1-Q3)], and the Mann-Whitney U test was performed. One-way ANOVA and post hoc Tukey’s test were performed to detect differences between multiple groups. Categorical factors were characterized by [n (%)] and analyzed by the χ2 test. ROC was performed to examine the diagnostic value, whereas the Youden index was conducted to define the threshold value of ROC. Data were evaluated using SPSS 22.0 and GraphPad Prism 6.0 for analysis. Bilateral *P* < 0.05 was illustrated as statistically meaningful.

## Results

### Demographic and clinicopathological data of the subjects

Table 1 presents the demographic and clinicopathological data of the subjects. No statistical differences in age, body mass index (BMI), gender, smoking, SBP, HDL-C, TG, TC, and UREA between ACS patients and controls (*P* > 0.05). However, the levels of DBP, LDL-C, creatine, white blood cell (WBC), and N-terminal pro-B-type natriuretic peptide (NT-proBNP) were typically higher in the ACS groups (*P* < 0.05).

### Upregulated serum miR-483-5p was positively associated with SYNTAX score and Gensini score

**Fig. 1:**
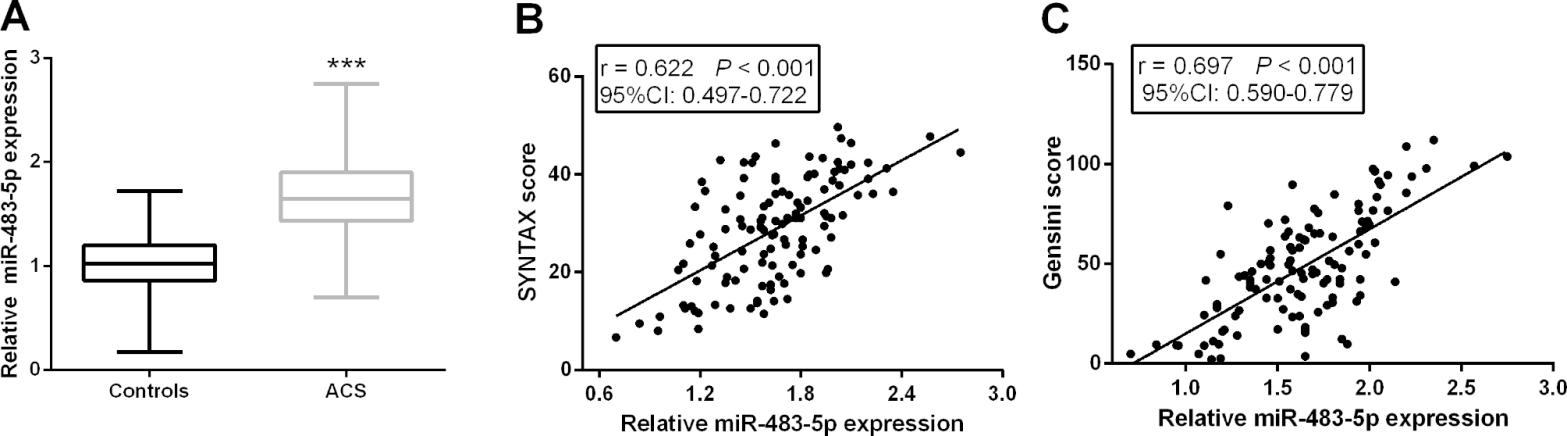
Serum miR-483-5p levels in ACS patients and correlation with different scores. **A**. Serum miR-483-5p levels of miR-483-5p in controls and ACS patients were explored by RT-qPCR. Pearson correlation coefficient was employed to evaluate the correlation of miR-483-5p with SYNTAX score (**B**) and Gensini score (**C**). *** *P* < 0.001 vs. Controls

Serum miR-483-5p in ACS patients before PCI was significantly higher than that in the controls (*P* < 0.01, Fig. 1A). Additionally, the SYNTAX score based on the anatomical structure was found to be associated with the severity of coronary lesions [19]. Pearson correlation coefficient analysis confirmed that miR-483-5p was positively associated with SYNTAX score (r = 0.622, *P* < 0.001, 95% CI: 0.497–0.722, Fig. 1B). Gensini score was established as a quantitative method for assessing coronary artery stenosis [9], and serum miR-483-5p was found to be positively associated with the Gensini score (r = 0.697, *P* < 0.001, 95% CI: 0.590–0.779, Fig. 1C**)**.

### Serum mir-483-5p has high diagnostic efficacy for ACS

**Fig. 2:**
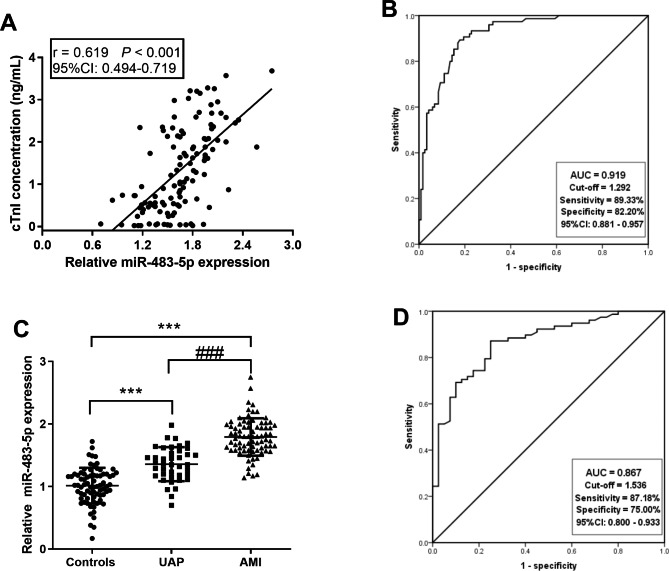
Diagnostic accuracy of serum miR-483-5p in patients with ACS. **A**. The correlation of miR-483-5p levels with cTnI concentrations in ACS patients. **B.** ROC based on miR-483-5p levels in controls and ACS patients. **C**. RT-qPCR detection of miR-483-5p levels in ACS patients with UAP and AMI. **D**. ROC based on miR-483-5p levels in UAP and AMI patients in the ACS group. *** *P* < 0.001 vs. Controls, ### *P* < 0.001 vs. UAP

Serum miR-483-5p levels were positively correlated with cTnI levels (r = 0.619, *P* < 0.001, 95%CI = 0.494 − 0.719), a common biomarker of ACS (Fig. 2A). The diagnostic performance of biomarkers is usually assessed by ROC. Figure 2B confirmed that the AUC of miR-483-5p was 0.919, and the sensitivity and specificity of differentiating ACS patients from controls were 89.33% and 82.20% at a cut-off value of 1.292, demonstrating a feasibility diagnostic value. RT-qPCR furthermore verified that miR-483-5p levels were elevated in both UAP and AMI compared to controls, and the AMI group was markedly elevated compared to the UAP group (*P* < 0.01, Fig. 2C). ROC also confirmed that the AUC for miR-483-5p to identify AMI patients from ACS patients was 0.867, and the sensitivity and specificity were 87.18% and 75.00%, respectively, when the cut-off value was 1.536 (Fig. 2D).

What’s more, the ACS and controls were treated as independent dichotomous variables in logistic analysis that included serum miR-483-5p levels and related clinical indicators. As presented in Table 2, miR-483-5p (OR = 9.664, 95%CI: 4.462–20.928, *P* < 0.001) and DBP (OR = 2.203, 95%CI: 1.053–4.609, *P* = 0.036) both independently contributed to the development of ACS.

**Table 2:** Relation of different parameters to the occurrence of ACS

Variables	OR	95% CI	*P* value
Age	0.703	0.323–1.527	0.373
Gender	0.491	0.233–1.035	0.062
BMI	0.698	0.324 − 1.503	0.358
Smoking	0.850	0.404–1.789	0.669
SBP	0.523	0.245–1.116	0.094
**DBP**	**2.203**	**1.053–4.609**	**0.036**
LDL-C	0.553	0.263– 1.161	0.117
HDL-C	0.919	0.419–2.013	0.832
TC	0.555	0.261– 1.181	0.127
TG	0.998	0.473– 2.107	0.995
Creatine	2.024	0.944–4.339	0.070
WBC	2.017	0.955–4.258	0.066
UREA	0.684	0.327–1.428	0.312
**MiR-483-5p**	**9.664**	**4.462– 20.928**	**0.000**

Note: BMI, body mass index; SBP, systolic blood pressure; DBP, diastolic blood pressure; HDL-C, high-density lipoprotein cholesterol; LDL, low-density lipoprotein cholesterol; TC, total cholesterol; TG, triglyceride; WBC, white blood cells

### Significance of serum mir-483-5p in predicting MACE post-PCI in ACS patients

**Fig. 3:**
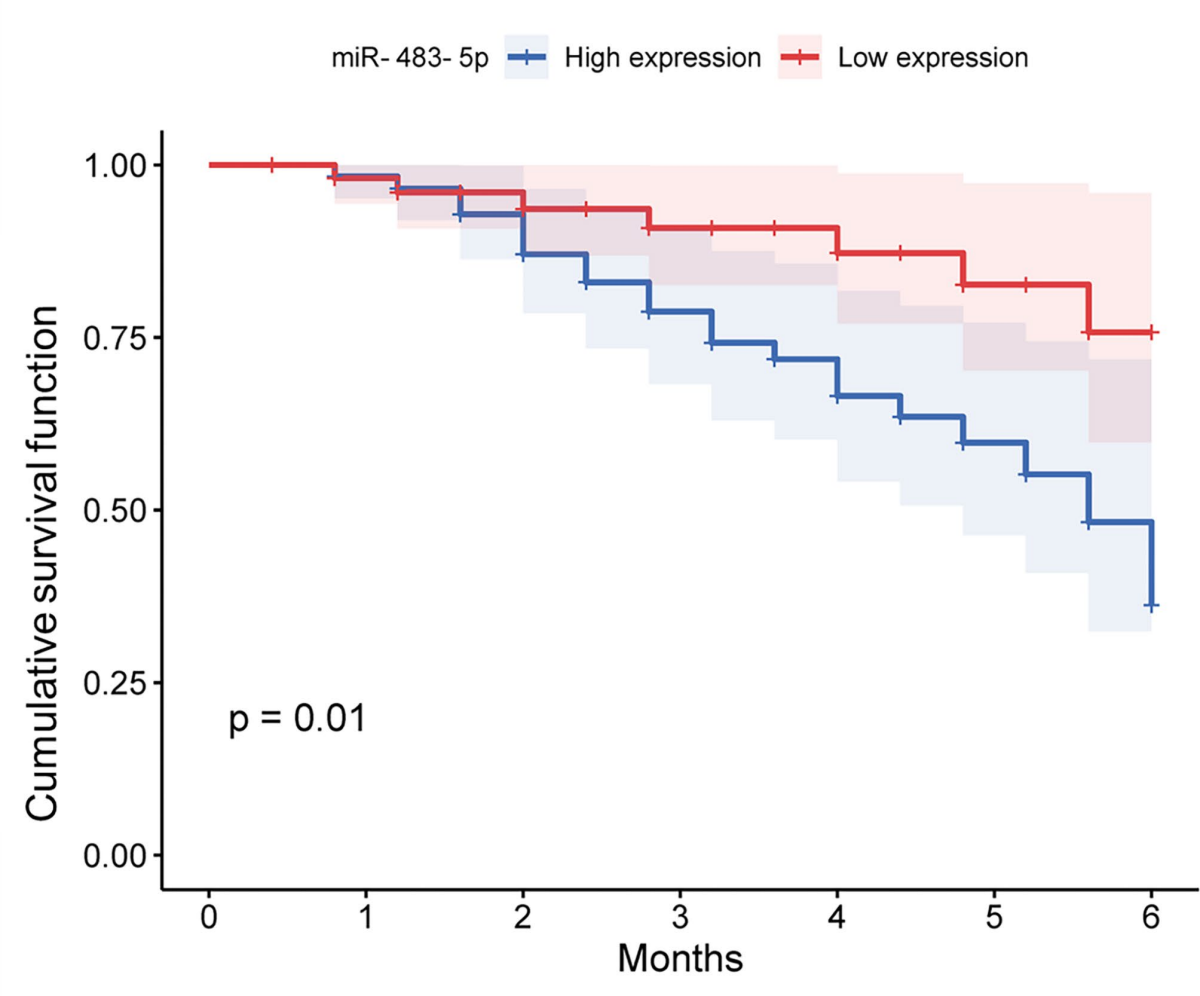
Kaplan Meier monitored the influence of miR-483-5p levels on ACS patients undergoing MACE during 6 months after PCI

**Fig. 4:**
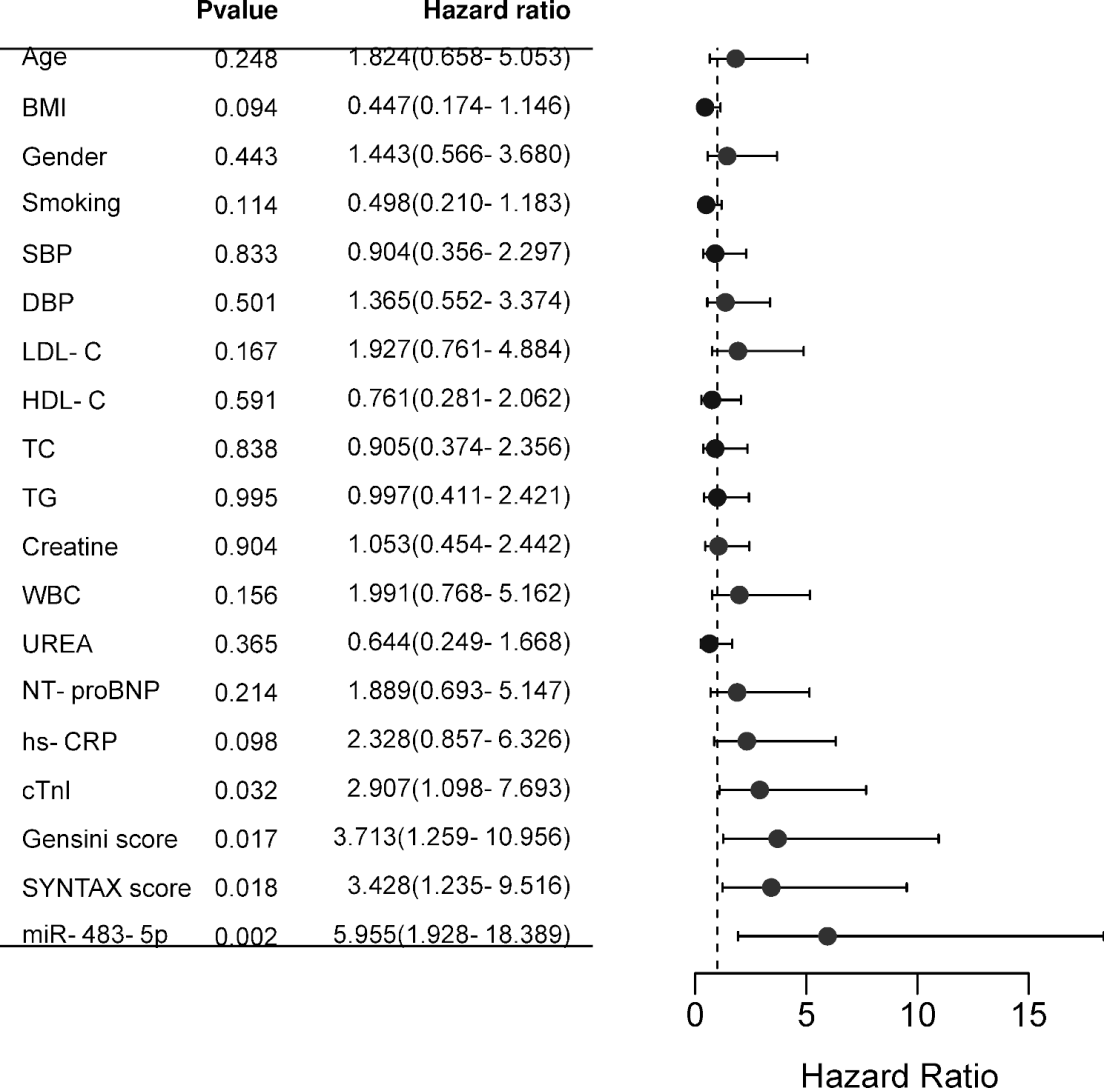
Cox regression analysis of potential influences factors affecting the occurrence of MACE after PCI in patients with ACS was performed and visualized with forest plots

During the 6-month follow-up period after PCI, the incidence of MACE was 24.58%, including 3 cardiac death, 6 revascularizations, 9 recurrent angina, 4 reinfarctions, and 7 heart failure. Divide ACS patients into high miR-483-5p group (n = 62) and low miR-483-5p group (n = 56) based on the mean miR-483-5p levels (1.64 ± 0.36). As shown in Table 3, high levels of miR-483-5p were significantly associated with LDL-C, HDL-C, NT-proBNP, cTnI, SYNTAS score, Gensini score, Baseline stenosis, and stent Diameter (*P* < 0.05). Furthermore, the high miR-483-5p level was found to be associated with a higher incidence of MACEs after PCI (*P* = 0.018, Table 4). Kaplan-Meier analysis confirmed the same results (*P* = 0.01, Fig. 3). COX regression analysis was performed as shown in Fig. 4, and similar to cTnI, Gensini score, and SYNTAX score, miR-483-5p (HR = 5.955, 95%CI: 1.928–18.389, *P* = 0.002) could be used as an independent predictor of MACE occurrence.

**Table 3:** Correlation of miR-483-5p levels with clinical information of ACS patients

Parameters	low miR-483-5p group (n = 56)	high miR-483-5p group (n = 62)	*P* values
Age, years	59.68 ± 8.24	58.02 ± 7.64	0.259
BMI, kg/m^2^	26.25 ± 4.09	27.225 ± 3.95	0.191
Gender, male, n (%)	30 (53.57)	38 (61.29)	0.489
Smoking, n (%)	33 (58.93)	31 (50.00)	0.318
SBP, mmHg	130.68 (123.75, 135.63)	128.70 (123.50, 136.62)	0.612
DBP, mmHg	82.20 (77.45, 87.10)	82.20 (74.98, 88.10)	0.983
LDL-C, mmol/L	2.70 (2.00, 3.05)	2.95 (2.38, 3.53)	**0.025**
HDL-C, mmol/L	1.38 (1.02, 1.68)	1.24 (0.83, 1.57)	**0.019**
TC, mmol/L	4.14 ± 0.49	4.08 ± 0.40	0.429
TG, mmol/L	1.23 ± 0.56	1.50 ± 0.57	0.437
Creatine, µmol/L	74.39 (68.62, 78.50)	73.25 (69.08, 78.21)	0.602
WBC, ×10^9^/L	7.42 ± 0.67	7.40 ± 0.75	0.859
UREA, mmol/L	4.92 ± 0.52	4.85 ± 0.49	0.479
NT-proBNP, pg/mL	102.74 ± 36.64	118.36 ± 34.63	**0.019**
hs-CRP, mg/L	5.82 ± 1.52	6.11 ± 1.26	0.255
cTnI, ng/mL	0.55 (0.28, 1.63)	0.71 (1.00, 2.38)	**0.000**
SYNTAX score	26.03 ± 10.60	30.83 ± 10.65	**0.016**
Gensini score	40.80 ± 22.13	55.96 ± 28.53	**0.002**
Culprit lesion, n (%)			
Left main	0 (0.00)	1 (1.61)	0.340
Left anterior descending	17 (30.36)	25 (40.32)	0.259
Left circumflex	10 (17.86)	19 (30.65)	0.107
Right	28 (50.00)	18 (29.03)	0.412
Baseline stenosis (%)	92.09 ± 3.70	95.81 ± 3.11	**0.000**
MVD, n (%)	34 (60.72)	39 (62.90)	0.807
Stent diameter (mm)	2.90 ± 0.48	3.21 ± 0.44	**0.003**
Stent length (mm)	25.86 ± 4.54	27.08 ± 4.36	0.139
Aspirin/clopidogrel, n (%)	7 (12.50)	11 (26.81)	0.429
ACE inhibitor/ARB, n (%)	15 (26.79)	16 (25.81)	0.904
β-blocker, n (%)	11 (19.64)	14 (22.58)	0.679
Calcium Channel blocker, n (%)	5 (8.93)	7 (11.29)	0.672
Stain, n (%)	5 (8.93)	13 (20.97)	0.069
Subgroups of ACS patients			
UAP/AMI	35/21	5/57	0.000

Note: ACE, angiotensin converting enzyme; ARB, angiotensin receptor blocker; BMI, body mass index; SBP, systolic blood pressure; DBP, diastolic blood pressure; HDL-C, high-density lipoprotein cholesterol; LDL, low-density lipoprotein cholesterol; MVD, multivessel disease; TC, total cholesterol; TG, triglyceride; WBC, white blood cells; hs-CRP, high-sensitivity C-reactive protein; cTnI, cardiac troponin I; Date was presented as mean ± SD, or median (first quartile, third quartile), or N (%)

**Table 4:** Major adverse cardiac events according to miR-483-5p expression levels

Variables	Cases No. (n = 118)	miR-483-5p expression		*P*
		High (n = 62)	Low (n = 56)	
Total MACEs	29 (24.58)	21 (33.87)	8 (14.29)	0.018
Death	3 (2.54)	2 (3.23)	1 (1.79)	0.538
Revascularization	6 (5.08)	5 (8.06)	1 (1.79)	0.129
Angina	9 (7.63)	7 (11.29)	2 (3.57)	0.108
Reinfarction	4 (3.39)	3 (4.84)	1 (1.79)	0.349
Heart failure	7 (5.93)	4 (6.45)	3 (5.36)	0.557

Note: Data are represented as n (%); MACE, Major adverse cardiac event

## Discussion

Current standard diagnostic methods for ACS included typical symptoms, ECG findings, and myocardial troponin levels[20]. However, in the emergency, ECG patterns diagnostic of ACS (insignificant ECG changes in the presence of persistent acute myocardial ischemia) are not present in some patients [21]. Moreover, myocardial troponin is not consistently elevated in AMI and lacks sensitivity in the first few hours due to its delayed release into the bloodstream, resulting in a “troponin blind period” [22]. As reported, the diagnostic sensitivity of cardiac troponin for myocardial injury within the first 3 h after admission to the emergency department is only 19–43% [23], and it can only be detected 6–12 h after coronary artery occlusion [24]. Additionally, myocardial troponin changes are common in chronic renal failure, acute pulmonary embolism, acute inflammatory myocarditis, and arrhythmias [25]. These pose significant obstacles to the diagnosis of ACS. To overcome this shortcoming, we have focused on miRNAs that are relevant to the pathogenesis of plaque rupture and can be easily detected and quantified.

As an endogenous RNA molecule that participated in the regulatory control of a range of developmental and physiological processes, miRNA dysregulation has been recognized as a useful marker for a wide range of diseases. This plays to the potential advantages of stable expression, ease of detection, and relevance to the clinicopathology of miRNAs. For example, miR-142-3p [6], miR-3464 [8], and miR-4286 [26] were identified as potential diagnostic or predictive biomarkers for ACS.

miR-483-5p, one of many miRNAs, has been suggested by several studies to have potential relevance to ACS. Firstly, coronary plaque rupture is the highest incidence of ACS, and Li et al. identified miRNAs significantly associated with plaque rupture, including miR-483-5p [11]. Atherosclerosis serves as the pathological foundation of ACS, and microarray analysis was conducted on both normal coronary arteries and arteries with plaque, where differential expression miRNAs included miR-483-5p [12]. Arrhythmia is a common complication of ACS, and miR-483-5p is involved in regulating atrial fibrillation, which is common in postoperative arrhythmias [13]. miR-483-3p, originating from the same precursor as miR-483-5p but located on the opposite arm of the pre-miRNA, exhibits consistent upregulation in heart failure patients with implanted left ventricular assist devices[27]. The results of previous studies indicate that miR-483-5p may be correlated with ACS, and in this preliminary study, we explore its diagnostic value for ACS. First, we evaluated miR-483-5p expression in 75 controls and 118 ACS patients. It was evidenced that miR-483-5p was typically elevated in ACS patients, which concurred with the results reported above. ROC is widely used to determine the accuracy of diagnostic biomarkers. Our results found that miR-483-5p has high sensitivity and specificity to identify ACS patients from controls. ACS was defined as AMI and UAP, and we also found higher levels of miR-483-5p in AMI patients than in UAP. And ROC established that serum miR-483-5p markedly distinguished AMI patients from UAP patients in ACS patients, demonstrating a high diagnostic potential.

PCI has become a common strategy for the treatment of ACS [28, 29], which can significantly restore coronary perfusion, reduce infarct size, and decrease cardiovascular mortality and disability [30], but some patients still develop MACE after performing PCI. SYNTAX score has been developed as an anatomy-based tool that can be used to define the complexity and progression of coronary artery disease and guide decision-making for PCI, as well as risk prediction for MACE [31]. miR-483-5p was confirmed to be positively correlated with the SYNTAX score. Additionally, the Gensini score, another widely used score for quantitative analysis of coronary lesions, is simpler and more scientific than the SYNTAX score and is more applicable to ACS patients treated with emergency PCI, enabling rapid assessment of coronary lesions and identification of high-risk patients, and timely treatment [18]. Our study also revealed a significant positive correlation between the Gensini score and miR-483-5p. Given the important role of miR-483-5p, we sought to explore the effect of miR-483-5p on MACE after PCI. To reflect the discrete profile of patients, we divided the patients into high miR-483-5p group and low miR-483-5p group based on their mean serum miR-483-5p values and found that the number of patients in the high miR-483-5p group was higher. In this preliminary study of ACS patients undergoing PCI with a 6-month follow-up, 29 patients experienced MACE, and most of them were patients with high miR-483-5p expression. Cox regression analysis confirmed that, together with cTnI, SYNTAX score, and Gensini score, miR-483-5p was an independent predictor of the experiencing MACE in patients after PCI. Finally, there are some limitations in this study. Because serum samples were collected from ACS patients at only time point, it was not possible to determine the time-dependent pattern of miR-483-5p expression in patients, which will be addressed in the next studies. New onset heart failure was determined by clinical signs and symptoms in a physical examination and on cardiac ultrasound and chest radiography. What’s more, multiple MACE events in a single patient were not identified in the follow-up MACE events due to the short follow-up time and small sample size, but we will expand the sample size and keep an eye on the occurrence of MACE events. Additionally, a significant positive correlation was observed between miR-483-5p and cTnI, a widely used biomarker for ACS. However, due to the lack of cTnI data from healthy individuals, this pilot study could not compare the diagnostic performance of cTnI and miR-483-5p in ACS patients. Collectively, our study determined the clinical diagnostic potential of miR-483-5p in patients with ACS, as well as its predictive accuracy for MACE after performing PCI.

## Data Availability

The datasets used and/or analyzed during the current study are available from the corresponding author upon reasonable request.
